# Clone Phylogenetics Reveals Metastatic Tumor Migrations, Maps, and Models

**DOI:** 10.3390/cancers14174326

**Published:** 2022-09-04

**Authors:** Antonia Chroni, Sayaka Miura, Lauren Hamilton, Tracy Vu, Stephen G. Gaffney, Vivian Aly, Sajjad Karim, Maxwell Sanderford, Jeffrey P. Townsend, Sudhir Kumar

**Affiliations:** 1 Institute for Genomics and Evolutionary Medicine, Temple University, Philadelphia, PA 19122, USA; 2 Department of Biology, Temple University, Philadelphia, PA 19122, USA; 3 Department of Biostatistics, Yale University, New Haven, CT 06510, USA; 4 Center for Excellence in Genomic Medicine Research, King Abdulaziz University, Jeddah 22252, Saudi Arabia; 5 Department of Ecology and Evolutionary Biology, Yale University, New Haven, CT 06525, USA; 6 Program in Computational Biology and Bioinformatics, Yale University, New Haven, CT 06511, USA

**Keywords:** tumor evolution, metastasis, molecular evolution, phylogenetics, phylodynamics, cancer

## Abstract

**Simple Summary:**

Metastasis is the spread of cancer cells across organs and is a major cause of cancer mortality. Analysis of tumor sequencing data provides a means toward the reconstruction of routes of metastatic cell migrations. Our reconstructions demonstrated that many metastases were likely seeded from pre-existing metastasis of primary tumors. Additionally, multiple clone exchanges between tumor sites were common. In conclusion, the pattern of cancer cell migrations is often complex and is highly variable among patients.

**Abstract:**

Dispersal routes of metastatic cells are not medically detected or even visible. A molecular evolutionary analysis of tumor variation provides a way to retrospectively infer metastatic migration histories and answer questions such as whether the majority of metastases are seeded from clones within primary tumors or seeded from clones within pre-existing metastases, as well as whether the evolution of metastases is generally consistent with any proposed models. We seek answers to these fundamental questions through a systematic patient-centric retrospective analysis that maps the dynamic evolutionary history of tumor cell migrations in many cancers. We analyzed tumor genetic heterogeneity in 51 cancer patients and found that most metastatic migration histories were best described by a hybrid of models of metastatic tumor evolution. Synthesizing across metastatic migration histories, we found new tumor seedings arising from clones of pre-existing metastases as often as they arose from clones from primary tumors. There were also many clone exchanges between the source and recipient tumors. Therefore, a molecular phylogenetic analysis of tumor variation provides a retrospective glimpse into general patterns of metastatic migration histories in cancer patients.

## 1. Introduction

Cancer is among the most prevalent complex diseases. More than 90% of cancer morbidity and mortality is caused by the spread (metastasis) of cancer cells from the original tumor to local and distant organs [1,2]. Existing metastases may also seed new tumors [3,4,5]. Tumor growth and metastasis occur over months to years, and most of the progression of metastatic clones is not detected until medical attention is needed [6,7]. It is rarely possible to trace the movement of cancer cells in patients over time [8]. However, advanced technologies and methods enable the mapping of metastatic migration histories retroactively: the record of the origination and diversification of tumor cells in a patient can be inferred by a comparative analysis of tumor genetic heterogeneity [9,10]. Cells in primary tumors and metastases undergo genetic changes, producing extensive intra- and inter-tumor heterogeneity that manifests in patient tumor samples [11,12,13]. The molecular evolution and spread of tumor cells parallel the molecular evolution and dispersal of species in evolutionary biology, where molecular phylogenetics has revealed the tempo and patterns of diversification of species and dynamics of their geographical distribution over time—even with a commonly scant fossil record [14]. A similar framework applied to the analysis of genetic variation found in tumors can reveal evolutionary relationships of cancer cells and clones within patients [14]. These evolutionary relationships are useful for inferring evolutionary, temporal, and spatial dynamics of metastatic cell migrations [15,16,17].

**Figure 1:**
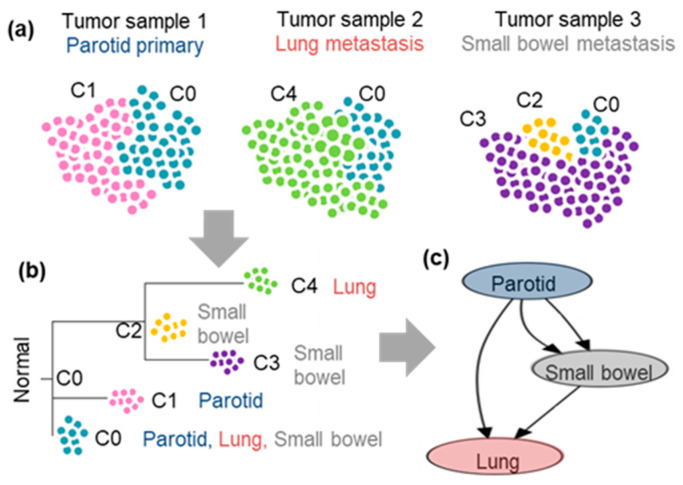
Inference of metastatic migration histories from a multi-sample bulk-sequencing dataset. (**a**) Patient ATP430 from Zhao et al. [6] was used. The data contained 5370 single-nucleotide variants. The primary tumor site was parotid. (**b**) CloneFinder [18] predicted clones C0–C4 (C0: blue, C1: pink, C2: yellow, C3: purple, C4: green). (**c**) Given CloneFinder clone phylogeny, PathFinder [9] inferred a metastatic migration map.

An analysis of a multi-sample sequencing dataset can reveal likely metastatic migration histories (Figure 1). For example, bulk sequencing of the primary tumor in the patient’s parotid gland and that of two metastases in their lungs and small bowel (Figure 1a) produced multi-sample genetic variation that was sufficient for computational deconvolution of clones and inference of the phylogeny not just of tumors [6], but of major clones (Figure 1b). Multiple clones could be detected in all three samples; some clones are shared between tumors (Figure 1b). Clone C0 is the most recent common ancestor (MRCA) of all the tumor clones found in all samples. Using the clone phylogeny with branch lengths capturing genetic evolution and the location of clones, a Bayesian reconstruction of the clone migration history suggests that both metastases were seeded by clones from the primary tumor (Figure 1c). A metastatic clone C2 also traveled from the small bowel to the lungs (Figure 1c). 

The development of computational methods to infer migration histories [9,10] and the sequencing of increasingly larger numbers of tumor biopsies from many patients [6,19,20] enables the evaluation of fundamental questions regarding the clonal history of cancers. For example, how frequently is a metastasis the source of seed clones for other metastases (M→M) compared to clones from primary tumors (P→M)? Do metastatic tumors often receive multiple clones from the source tumors? Furthermore, how often do tumors engage in a reciprocal exchange of clones (M↔M and P↔M)?

Here we address these questions by analyzing tumor bulk-sequencing data from 51 metastatic cancer patients. This investigation complements previous insights from metastatic seeding events and migration histories from bulk-sequencing of just one or two tumor sites, for which the clonal deconvolution is difficult [21]. Additionally, it is complementary to numerous other individual studies that were not yet in a position to leverage modern computational approaches or that analyzed only a few patients or cancer types [22,23,24,25,26]. We analyze multi-tumor site sequencing data from many patients to generate global insights into the clonal evolution of cancer. We unravel the complexity of cancer cell migration routes from evolutionary analyses of tumor variation dataset and evaluate alternative metastatic progression models [4,5,27,28], contrasting patterns across cohorts.

## 2. Materials and Methods

### 2.1. Data Collection

We obtained datasets from supplementary information from three published articles: Zhao, Hu, and De Mattos cohorts [6,19,20]. In the Zhao cohort, datasets contained genetic information for the primary tumors and at least two metastatic sites. The primary tumor tissues were from the bladder, breast, cervix, cholangiocarcinoma in liver, colon, endometrium, head and neck, lung, ovary, pancreas, renal clear cell carcinoma, sarcoma, and spinal. The datasets from the Hu cohort consisted of metastatic colorectal cancer samples. The metastatic breast cancer patients who underwent post-mortem warm autopsies were in the De Mattos cohort.

These datasets were generated through bulk sequencing (exome sequencing). We obtained all single-nucleotide variants (SNVs) identified and processed in the original studies. For each SNV, these datasets contained total read counts and counts of reads with altered bases. Similarly, we obtained the information on tumor sites for each sample from their supplementary information. We excluded datasets composed of only two tumor samples because deconvolution of full clone genotypes is not reliable [29]. Additionally, one of our goals was to investigate how often a metastatic tumor is seeded from another metastatic tumor, for which datasets with only two tumor samples would bias the results. 

### 2.2. Reconstruction of Clone Phylogenies

We inferred clone genotypes, their phylogenetic relationship, and clone frequencies for each dataset using CloneFinder [18], one of the best-performing methods for clone genotype prediction and clone phylogenetic inferences from tumor population bulk sequencing data [29]. In the CloneFinder analysis, we used all SNVs identified in the original studies, including pathogenetic and non-pathogenetic mutations. We only used the observed read counts when the total read count was greater than 40 and the variant read count was greater than six because the inclusion of SNVs with low read counts potentially affects clone frequency estimation. Tumor purity was estimated by summing up inferred tumor clone frequencies for each sample. Branch lengths in an inferred clone phylogeny were calculated using a maximum parsimony approach in MEGA [30,31]. Note that this approach does not need or use the information on the timing of tumor sampling. 

### 2.3. Inference of Tumor Migration Histories

We reconstructed a metastatic migration history for each inferred clone phylogeny using PathFinder [9], chosen because it had achieved a higher accuracy in comparison to other methods (e.g., MACHINA [10]), as shown in previous studies [8,15,16]. We assumed a clone was present in a tumor site when its inferred clone frequency by CloneFinder was greater than zero. In the PathFinder analysis, we used the default settings, because a benchmarking study showed that the default settings performed well [9]. Following the default setting, migration paths predicted with <0.5 posterior probability were removed. For these filtered migration paths, all paths with >0.15 posterior probability were collected, and the most parsimonious path was selected. Up to thirty possible polytomy resolutions were tested, but 100 alternative resolutions were tried for the ATP456 dataset because it contained many polytomies in the clone phylogeny. Due to the greater number of polytomies, we explored 1000 alternative phylogeny resolutions for two other datasets (ATP429 and ATP442). All De Mattos cohort datasets lacked the primary tumor information, so we performed the PathFinder analysis assuming that the MRCA clone (C0 in Figure 1) was present in the primary tumor site. Under this assumption, no reciprocal clonal exchanges with primary tumors could be predicted. PathFinder inferences for two datasets from the De Mattos cohort (patients 308 and 315) had low overall posterior supports for inferred migration paths (<0.3), so we removed these two datasets from the analysis. In total, the number of patients analyzed was 33, 12, and 6 for Townsend, Hu, and De Mattos cohorts. 

## 3. Results and Discussion

### 3.1. Clonal Composition of Tumors 

**Figure 2:**
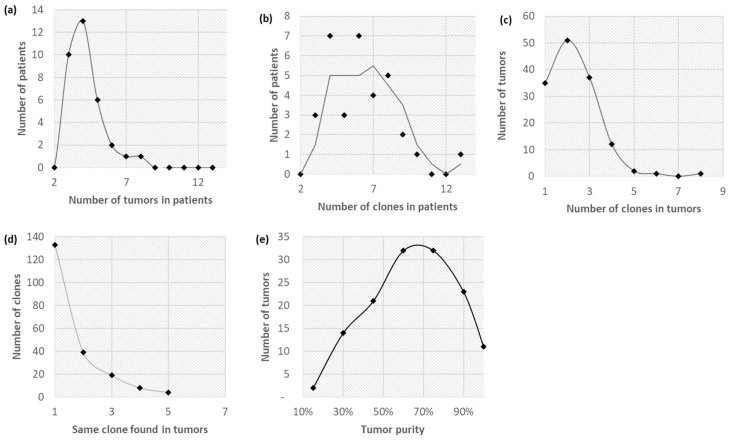
The distribution of clones in 139 tumors sampled from 33 patients from the Zhao cohort, deconvolved into 203 clones. (**a**) The distribution (smoothed line) of the number of distinct tumors in patients (mean: 4.2, median: 4). (**b**) The distribution (sliding-average line with a window of 2) of the number of distinct clones in patients (mean: 6.2, median: 6). (**c**) The distribution (smoothed line) of the number of clones in tumor samples (mean: 2.3, median: 2). (**d**) The distribution (log-transformed fit line) of the number of tumor samples in which each clone was found (mean: 1.6, median: 1). (**e**) The distribution (smoothed line) of tumor purities (estimated from the clonal composition of tumor samples; mean and median tumor purity were both 61%).

We analyzed the Zhao et al. [6] dataset (Zhao cohort; 33 patients) suffering from various metastatic cancers. We followed the analysis protocol in Figure 1 for each multi-sample bulk-sequencing dataset, which produced clone genotypes, phylogeny, and migration history for every patient. Tumors of patients in the Zhao cohort exhibited extensive clonal diversity. The number of distinct tumors (Figure 2a; median: 4) and clones (Figure 2b; median: 6) varied extensively across patients in the Zhao cohort. Twenty-five percent of the tumor samples were monoclonal, and thirty-eight percent of the tumors contained more than two clones (Figure 2c). Two-thirds of the clones were found in only one tumor, and 15% of the clones were present in three or more tumor samples (Figure 2d). In fact, a patient generally contained at least one polyclonal metastatic tumor. Tumor purity ranged across a spectrum, averaging 61% (Figure 2e).

### 3.2. Clone Phylogenies

**Figure 3:**
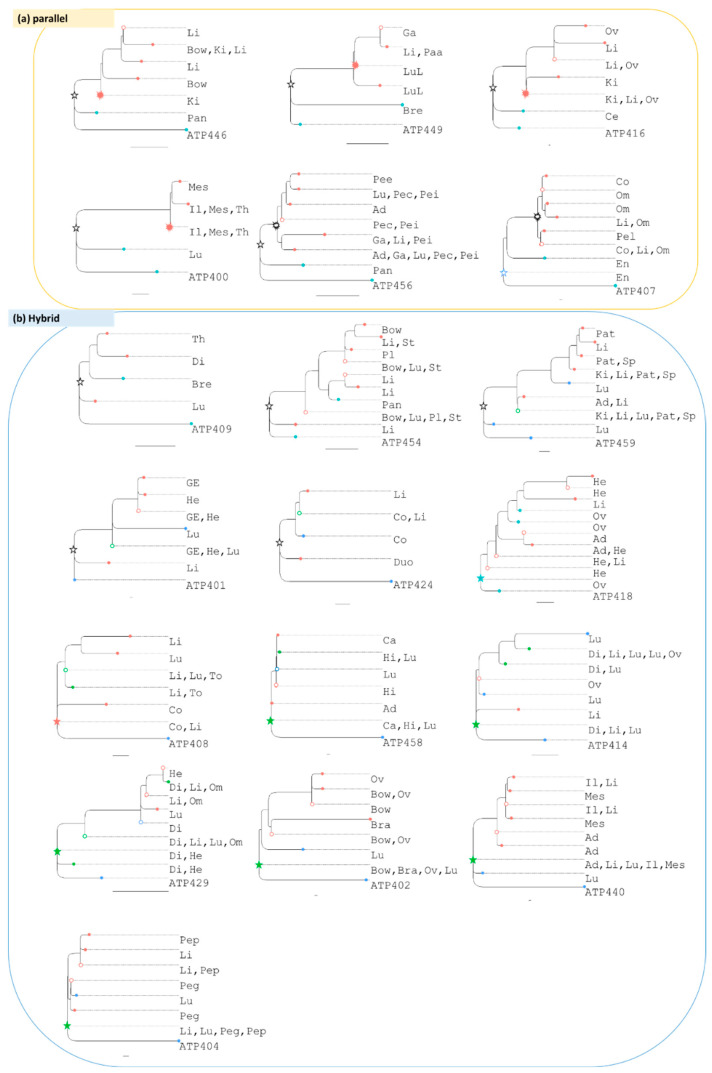
Phylogenies from the Zhao cohort. Inferred phylogenies were rooted with normal cell sequence (patient ID shown next to the normal cell) and classified into the parallel (**a**) and hybrid progression models (**b**), based on the shape of the phylogenies and the locations of clones (Blue and red tips: clones identified only within the primary and metastatic tumors, green tips: clones identified in both primary and metastatic tumors; open circle: ancestral clone, star: most recent common ancestor [MRCA], starburst: MRCA of metastatic clones [meta-MRCA], black star and starburst: MRCA and meta-MRCA clones that are not found in any tumors). Tumors that contain a clone are listed next to each tip. Below is the abbreviation used for each tumor site: Adrenal (Ad), Bladder (Bl), Bone (Bon), Bowel (Bow), Brain (Bra), Breast (Bre), Carinal LN (Ca), Cervix (Ce), Colon (Co), Diaphragm (Di), Duodenum (Duo), Dura (Dur), Endometrium (En), Esophagus (Es), Gallbladder (Ga), GEJ (GE), Heart (He), Ileum (Il), Hilar LN (Hi), Ileal wall (Il), Kidney (Ki), Liver (Li), Lung (Lu), Lung LN (LuL), Lung pleura (Lup), Lymph node (Ly), Meninges (Men), Mesentery (Mes), Omentum (Om), Ovary (Ov), Pancreas (Pan), Paraaortic LN (Paa), Paratrachael LN (Pat), Parotid (Par), Pelvic peritone (Pel), Pericardium (Pec), Perigastric LN (Peg), Perihepatic LN (Pee), Perihilar LN (Pei), Peripancreatic LN (Pep), Peritoneum (Pet), Pleura (Pl), Small bowel (Sm), Spleen (Sp), Stomach (St), Thyroid (Th), and Tongue (To). Only datasets with primary tumor sequences and at least three metastatic tumor sites are included. Scale bar: ten mutations.

Clone phylogenies for the patients in the Zhao cohort were distinct for each patient (Figure 3). The most recent common ancestor (MRCA) of all observed tumor clones was at the root of the clone phylogeny (star symbols; Figure 3). The MRCA clone was found in at least one primary tumor in eight patients and in at least one metastatic tumor in seven patients. In six patients, the MRCA clone was observed in both primary and metastatic tumor sites, e.g., patient ATP440′s primary and three metastases contain the MRCA clone. In contrast, patient ATP408′s MRCA clone was found only in the metastatic tumors, suggesting that it is present at too low frequencies to sample or went extinct in the primary tumor. Many other ancestral clones (open circles) were also found in primary and metastatic tumor sites in most phylogenies. The co-occurrences (green circles) are indications of clone migrations and exchanges between tumors.

**Figure 4:**
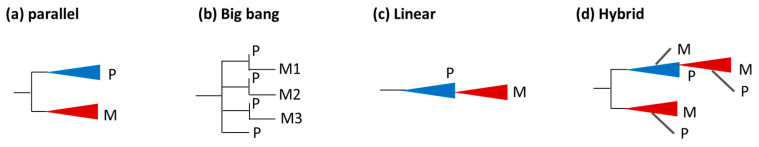
Progression models and the shapes of phylogenies. Expected shapes of phylogenies of tumors (P: primary and M: metastatic) for (**a**) parallel, (**b**) “big bang”, (**c**) linear, and (**d**) hybrid models.

In six phylogenies, all the clones found in metastatic tumors formed a monophyletic group, i.e., descended from a common ancestor (starburst marks). This monophyletic group is most closely related to a clone in the primary tumor (Figure 3a). The most parsimonious explanation for this pattern is that a clone from the primary tumor seeded a metastasis, which likely seeded other metastases because the metastasis clone phylogeny shows a significant branching pattern. These clone phylogenies are neither consistent with a linear progression model [4,27], which would require ancestor–descendant relationships between clones (Figure 4c), nor a big-bang model [28], which is expected to result in a star phylogeny (Figure 4b). Instead, the shapes of these phylogenies suggest that clones in primary and metastatic tumors evolve continuously, consistent with a parallel progression model [4,27] (Figure 4a).

However, most clone phylogenies are not consistent with a purely parallel progression model either (Figure 3b). This inconsistency with parallel progression arises because of seeding by different subsets of clones from respective primary tumors. Thus, in most phylogenies, neither primary nor metastatic tumor clones are monophyletic, and most clone phylogenies show a metastatic progression that is a mix of linear and parallel patterns (Figure 4d). Additionally, some primary clones are nested within clades of metastatic clones (e.g., ATP409), indicating seeding to primary from a metastatic tumor (self-seeding) [5].

Because of these trends and frequent clone sharing between primary and metastatic tumor sites (including ancestral and MRCA clones; green tips and nodes in Figure 3b), clonal migration histories among tumors cannot be easily delineated through visual inspection of clone phylogenies. Therefore, we applied computational methods using clone phylogenies and clone locations to infer the statistically most likely migration paths.

### 3.3. Tumor Migration Histories

**Figure 5:**
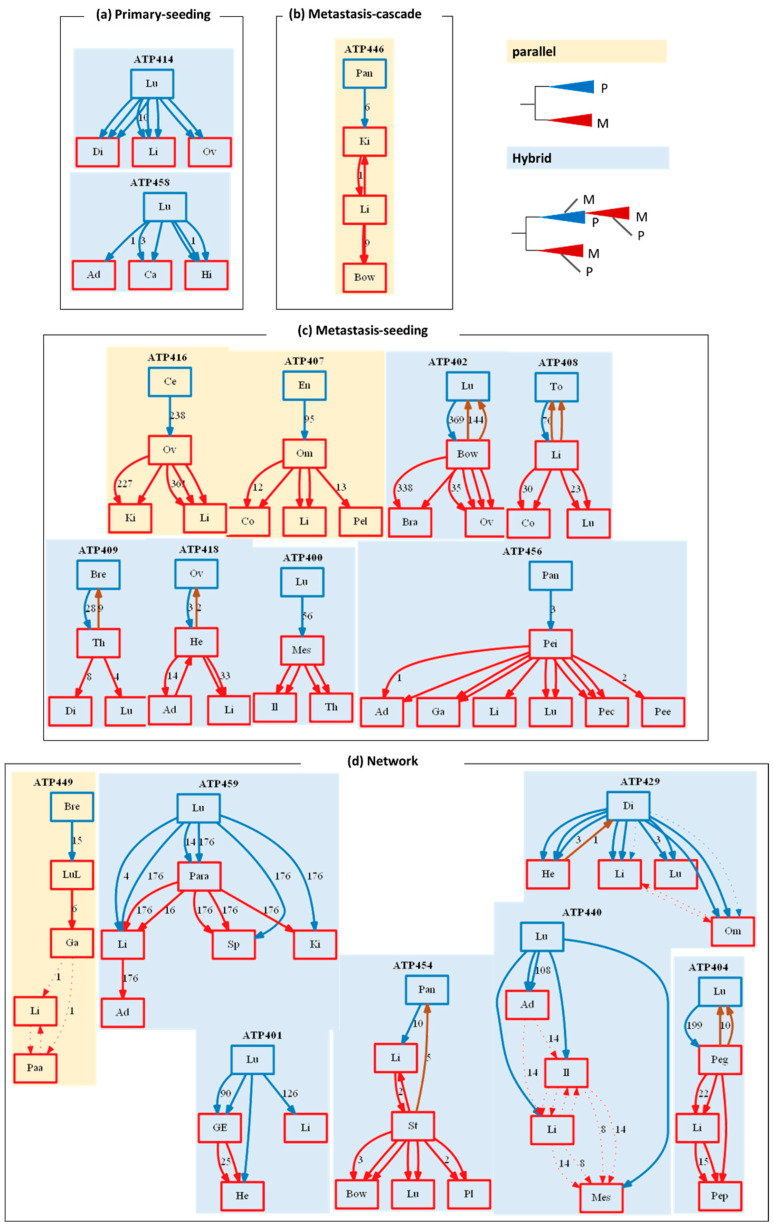
Metastatic migration histories inferred from clone phylogenies in Figure 4. The migration maps for each patient ID (top) were classified into seeding models based on their shapes (**a**–**d**). The number of variants mapped is shown next to a path (solid: high support, dashed: low support, blue: P→M, red: M→M, brown: M→P) between sites (Primary: blue and metastatic: red) when their count is greater than zero. An abbreviation (Figure 4 legend) for each tumor site labels each box.

Tumor migration histories were inferred (see Section 2) for patients with a primary tumor and at least three metastatic tumor sites in the Zhao cohort (Figure 5). All the metastases were seeded directly by clones from the primary tumors in two patients, which is consistent with the seeding hypothesis, where all metastases are seeded by primary tumor clones [4] (Figure 5a). Two or more different clones from the primary tumor seeded different metastases in these patients, as each arrow points to the migration of a clone between tumor sites. Additionally, many clones in metastases are shared with the primary tumors, indicating that new mutations have not occurred for the founder clones in metastatic tumors. The other founder clones in metastatic tumors show a few base differences from the seed clone in the primary tumor, e.g., ten sequence differences for lung and liver clones in patient ATP414.

Another patient exhibited a metastatic cascade in which the primary tumor seeded only one of the metastases. The rest of the metastases were seeded by other metastases in a linear progression [4,5] (Figure 5b). But in many of the migration maps, clones from the primary tumor seed an initial metastasis that then seed other sampled metastases (Figure 5c). That is, a pre-existing metastasis spreads to form new metastases. A more complex seeding scenario was observed in about half of the migration maps, in which clones founded some tumors from more than one source tumor (Figure 5d). Overall, complex seeding patterns were the most common in the Zhao cohort.

### 3.4. Preponderance of Each Type of Clonal Migration and Exchange

**Figure 6:**
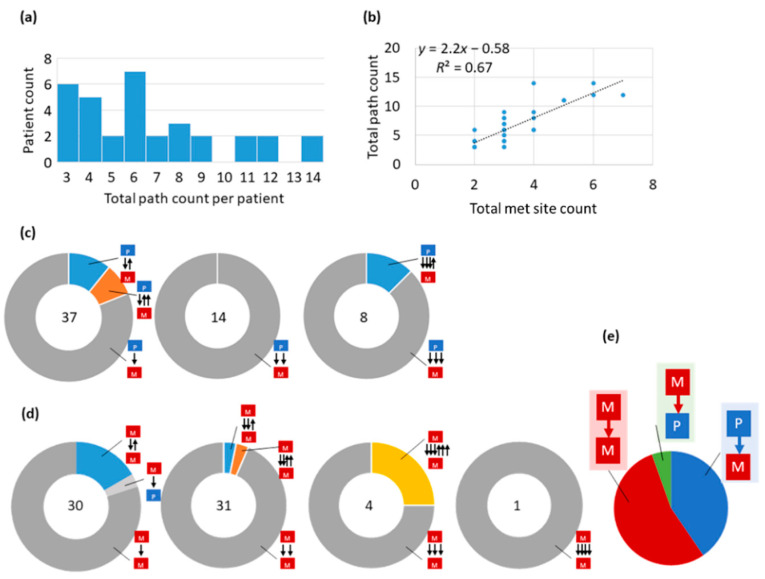
Distribution of clone migrations for the Zhao cohort dataset. (**a**) Histogram of migration path counts in patients. (**b**) Linear regression of the number of migrations against the number of metastatic sites sampled. (**c**) The number (center) and proportion of migrations of clones from primary tumors. Each black arrow indicates a seeding event. The direction of an arrow represents the orientation of the seeding event. Multiple arrows with the same direction indicate polyclonal seeding events, where the number of arrows corresponds to the number of clones migrated. (**d**) The number (center) and proportion of migrations of clones from metastatic tumors of tumor pairs. (**e**) Proportions of path types. To infer seeding events, we first built clone phylogenies from sequence data using CloneFinder, and then each clone phylogeny was analyzed using PathFinder.

Edges in the migration maps (Figure 5) often correspond to more than one migration path, suggesting that multiple clones have migrated from one tumor site to another (polyclonal seeding). It is not possible to discern if multiple clones moved together; therefore, we tallied each clone migration as an independent event (asynchronous seeding, [4,5,27]) to summarize their counts. The total clone migrations (seeding events) vary across patients ranging from 4 to 14 paths (Figure 6a), with this total positively correlated with the number of sampled tumor sites (Figure 6b). 

The number of M→M paths for the whole dataset is slightly higher than P→M paths (Figure 6e). The number of self-seeding events (M→P) is also noteworthy, but their number is much smaller than that of P→M or M→M paths. A single clone migration (monoclonal seeding) was the most common type of primary to metastatic migration (63%; Figure 6c). Still, migrations of two clones (polyclonal seeding) were as common as monoclonal seeding between metastatic tumor sites (Figure 6d). Reciprocal clonal exchanges were also observed between primary and metastatic tumor sites (10%) and between metastatic tumor sites (14%; Figure 6c,d). The reciprocal clonal exchanges can also be of polyclonal origin, i.e., more than one clone has migrated back to the source site. For example, two reciprocal clonal exchanges were reconstructed from the metastatic-to-primary site for patients ATP402 and ATP408 (Figure 5). 

We also reconstructed migration histories for the De Mattos cohort [19] (six patients with primary breast tumors) and Hu cohort [20] (twelve patients with primary tumors in the colon) because these datasets contain patients sampled for more than one sample at each tumor site—that facilitates the capture of clonal diversity with a higher resolution than the Zhao cohort. Indeed, the number of clones within tumor sites was larger for De Mattos and Hu cohorts than the Zhao cohort for the patient with breast and primary colorectal tumors, respectively.

**Figure 7:**
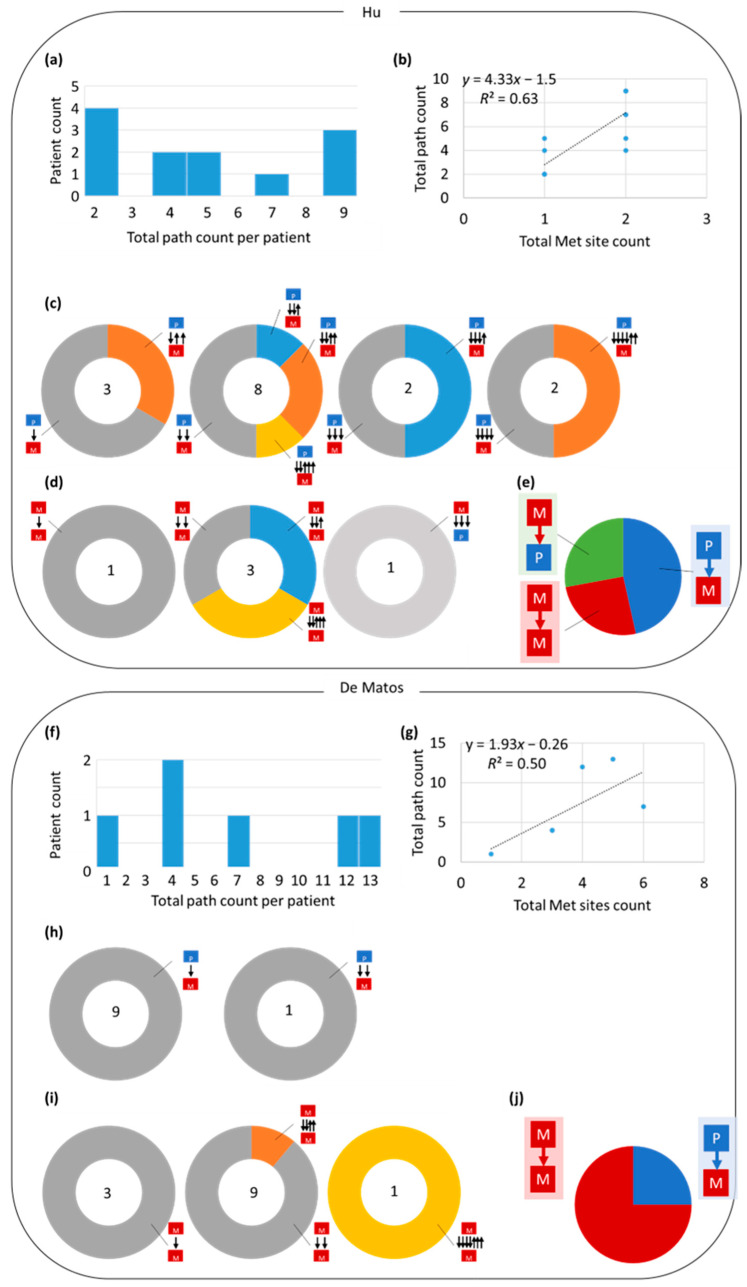
Distribution of clone migrations for the (**a**–**e**) Hu and (**f**–**j**) De Mattos cohort datasets (**a**,**f**) Histograms of migration path counts in patients. (**b**,**g**) Linear regression of the number of migrations against the number of metastatic sites sampled. (**c**,**h**) The number (center) and proportions of migrations of clones from primary tumors. (**d**,**i**) The number (center) and proportions of migrations of clones from metastatic tumors. (**e**,**j**) Proportions of path types. The De Mattos cohort does not contain primary tumor data, thus there are no M→P paths to infer.

Overall patterns for the Hu cohort were qualitatively similar to those for the Zhao cohort (Figure 7a–e). However, the frequency of M→M paths was lower than the P→M paths, whereas M→P frequency was relatively high in the Hu cohort (Figure 7e). Since the Hu cohort tended to have a smaller number of metastatic tumor sites per patient than the Zhao cohort, the Hu cohort did not have much power to detect M→M paths. The lower proportion of M→M paths occurred partly because the number of paths detected was directly related to the number of tumor sites sampled (Figure 7a,b). Additionally, polyclonal seeding and reciprocal exchanges were observed (Figure 7c,d). However, these exchanges were more common in the Hu cohort than in the Zhao cohort. The higher exchange rate probably arises because of the larger number of samples per tumor site in the Hu cohort.

Results from the De Mattos cohort exhibited the same pattern as the Hu cohort (Figure 7f–j). The De Mattos cohort had larger frequencies of reciprocal exchanges than the patients with primary breast tumors in the Zhao cohort, potentially due to the larger number of samples per tumor. Therefore, the Zhao cohort did not have enough power to detect many polyclonal seeding and reciprocal exchanges. Many more M→M paths were identified in the De Mattos cohort (Figure 7j), suggesting that self-seeding and cross-metastatic seeding are not necessarily rare.

## 4. Conclusions

We have reconstructed patient clone phylogenies and metastatic migration histories and demonstrated that tumor molecular evolution analyses could ascertain patterns of tumor genetic heterogeneity and clonal exchanges between tumor sites. Overall, our analysis of phylogenies indicated that migration histories generally do not follow any single model but rather a mixture of linear, parallel, and “big-bang” style progressions (68% of the Zhao cohort patients). Therefore, the pattern of cancer progression is often complex. Similarly, inferred metastatic migration histories are often complex, as tumors often received more than one clone from different tumor sites (network model; 41%). All patients classified into the network migration model followed the hybrid cancer progression model, except for one patient. Therefore, cells frequently migrate to other tumor sites, founding new cancer cell populations at different tumor sites. Additionally, we found as many polyclonal seeding events as monoclonal seedings, indicating that multiple clones have migrated from one tumor site to another. The observation of many polyclonal seeding events possibly supports the occurrence of multiple distinct clones migrating together, although our analysis cannot distinguish between these possibilities. Lastly, reciprocal seeding events occur at a small but appreciable frequency, resulting in a hybrid cancer progression in many patients. 

In previous studies, the patterns of metastatic seeding events and cell migration histories have been investigated by sequencing just one or two metastatic tumor sites together with their primary tumors, focusing on the initial and early metastatic events [21]. Although some other studies included secondary and subsequent metastatic events by analyzing more than two metastatic tumors, the number of patients in these studies was limited, preventing a general understanding of the pattern of metastatic cell migration events [22,23,24,25,26]. Therefore, our study is complementary to these previous studies, as we have analyzed many metastatic tumors from many patients. 

A limitation of our study is that the resolution of inferred clone phylogenies is potentially low as only a single sample was sequenced for each tumor site in the Zhao cohort. As a result, the number of migration events is potentially underestimated. In fact, the number of inferred polyclonal seeding events was larger for the Hu cohort, which sequenced more than one section for each tumor site. Additionally, inferred phylogenies are often affected by polytomies, and clones are shared by more than one tumor site, causing difficulties in the inference of migration histories, e.g., the low posterior probability of the inference (see Section 2). Sequencing more tumor sections potentially alleviates these issues. Another limitation is that the number of patients for each cancer type is too small to compare the patterns between them. Since several other studies have begun sequencing many samples for each patient, it is interesting to compare migration models and patterns between cancer types and other patients’ characters (e.g., treatments) in the future.

In the future, we expect the application of our protocol to more datasets with higher genomic coverage to provide more detailed information on the relative preponderance of different types of migrations and exchanges. We will be able to discern clones more accurately, infer clone phylogenies and resolve polytomies in the clone tree, and establish paths more effectively. Data from many more patients for each cancer type will also enable us to contrast different modes of migrations. If collected within a prospective cohort study or within a meticulously controlled high-sample retrospective analysis, clonal heterogeneity, migration paths or mutational frequency could be compared for prognosis, outcomes of treatment, or survival. Overall, molecular phylogenetic analysis of tumor variation provides some systematic answers to fundamental questions about metastatic migration.

## Data Availability

The data used in this study are available at supplementary information from published articles [6,19,20].
